# Rapid quantitative PCR equipment using photothermal conversion of Au nanoshell

**DOI:** 10.1038/s41598-024-54406-0

**Published:** 2024-02-16

**Authors:** Jae Sung Ahn, Woongkyu Park, Do Hyun Jeong, Soo Bong Choi, Sun Hee Ahn

**Affiliations:** 1 Bio and Health Photonics Research Center, Korea Photonics Technology Institute, Gwangju, South Korea; 2 Photonic Energy Components Research Center, Korea Photonics Technology Institute, Gwangju, South Korea; 3 Department of Physics, Incheon National University, Incheon, South Korea

**Keywords:** Biophotonics, Optical metrology, Nanoparticles

## Abstract

The emergence of infectious diseases worldwide necessitates rapid and precise diagnostics. Using gold nanoshells in the PCR mix, we harnessed their unique photothermal properties in the near-infrared regime to attain efficient heating, reaching ideal photothermal PCR cycle temperature profile. Our photothermal PCR method expedited DNA amplification while retaining its detection sensitivity. Combining photothermal quantitative PCR with real-time fluorometry and non-invasive temperature measurement, we could amplify the target DNA within just 25 min, with a minimum detectable DNA amount of 50 picograms. This innovation in photothermal qPCR, leveraging the photothermal properties of gold nanoshells, will pave the way for immediate point-of-care diagnostics of nucleic acid biomarkers.

## Introduction

The worldwide epidemics of infectious diseases have created an urgent need for rapid point-of-care diagnostics, but the thermal cycling process of conventional PCR is still time-consuming and resource-intensive, limiting its use in point-of-care diagnostics. As alternatives to conventional PCR, molecular diagnostic technologies such as Loop Mediated Isothermal Amplification (LAMP)^1,2^, microfluidic PCR^3–5^, and photothermal PCR^6,7^ have been proposed. Among these methods, photothermal PCR has attracted significant attention due to its ability to deliver fast diagnosis results by converting absorbed light energy into thermal energy, enabling faster thermal cycling^7–9^. Although photothermal PCR ensures rapid diagnostics, quantifying the results through fluorescence measurement is essential for its application in point-of-care diagnosis. With the emergence of photothermal quantitative PCR, there has been a surge in research, as it introduces a capability for quantitative analysis of infectious disease-associated factors, surpassing the limitations of previous photothermal PCR methods^6,10–16^.

The two primary methods for implementing photothermal quantitative PCR are: (1) blocking the photothermal light source to prevent its reach to the fluorescence detector, and (2) distinguishing the wavelength of the photothermal light source from that of fluorescence excitation/detection. The first method's downside is the necessity to restrict the volume of the PCR sample chamber to ensure efficient heat transfer beyond the structure that blocks the photothermal light source. The second method employs materials with high near-infrared absorption as photothermal conversion substances to avert interference with fluorescent signals in the visible light band. Researchers are actively exploring materials with excellent photothermal properties in the near-infrared spectrum, such as gold nanostructures^6,12,17,18^, graphene oxide (GO)^11,19^, and two-dimensional nanomaterials^20,21^, to broaden their biological applications. Gold nanostructures, which produce a photothermal effect through plasmonic resonance, offer easier control over the absorption resonance wavelength^18,22^. The photothermal effect in gold nanostructures is driven by a sequence of mechanisms including surface plasmon resonance, Landau damping, carrier relaxation, and thermal dissipation. This process leads to a rapid increase in ambient temperature within a timescale of just a few to tens of nanoseconds, facilitating extremely rapid thermal cycling^23,24^. They can be mass-produced with consistent quality and are thus commonly used in various photothermal qPCR studies^6,12^.

In this study, we developed novel photothermal quantitative PCR (qPCR) equipment, leveraging the rapid plasmonic heating effects of gold (Au) nanoshells. Comparing the time required for our photothermal PCR method using lambda DNA as a template with conventional PCR techniques revealed significantly faster amplification, affirming the efficiency and potential of our approach. Additionally, the detection sensitivity of the photothermal PCR method was confirmed using electrophoresis imaging, complemented by an analysis of the qPCR’s threshold cycle (C_t_) values and standard PCR curves.

## Methods

### Photothermal qPCR setup

**Figure 1:**
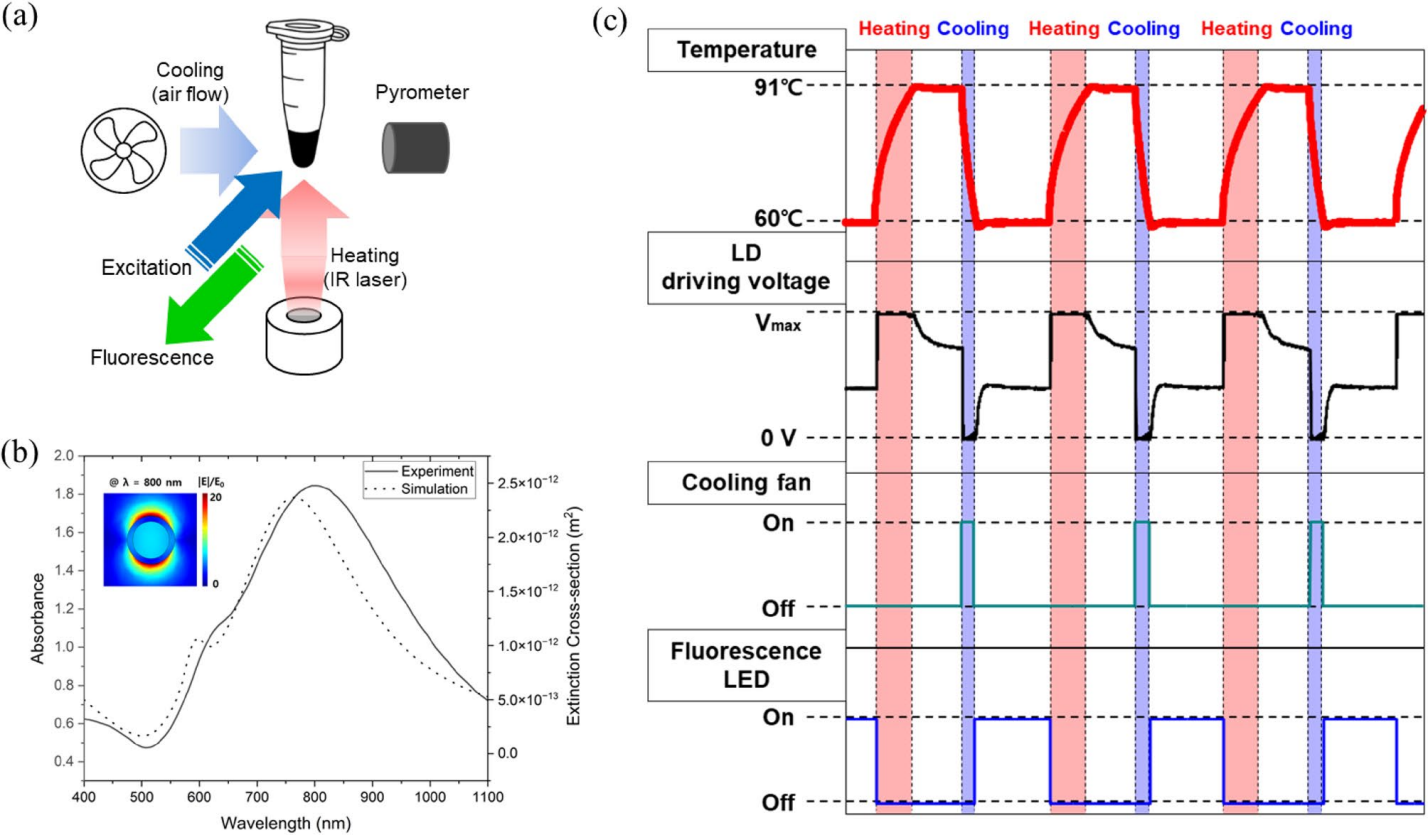
(**a**) Schematic of photothermal quantitative PCR setup. (**b**) Absorption spectrum of Au nanoshell solution (solid line: experiment, dotted line: simulation). The inset shows electric field enhancement due to plasmonic resonance of Au nanoshell at λ = 800 nm. Inset: Simulation results of electric field distribution around a gold nanoshell at a wavelength of 800 nm. (**c**) Operation timing and control method of the main components according to the photothermal PCR cycle progression.

Figure 1a illustrates the schematic of the photothermal qPCR setup. A focused laser (MDL-III-808, Changchun New Industries Optoelectronics Tech. Co., Ltd., Changchun, P. R. China) beam (λ = 808 nm, Output Power = 2.5 W) was directed from the bottom onto the PCR tube, facilitating photothermal heating of the Au nanoshells. Since contact temperature sensors could inhibit PCR by adsorbing reagents such as polymerase^25,26^ (Supplementary Fig. S1), the PCR mixture's temperature was monitored non-invasively using a pyrometer (CTLaserLTCF1, Optris GmbH, Berlin, Germany) (Supplementary Fig. S2). For calibration, a K-type thermocouple (TT-K-40-36, Omega Engineering, Norwalk, Connecticut) was used to align the temperature of the PCR mixture with the pyrometer's readings prior to the experiment. The PCR mixture's temperature was regulated by a PID feedback algorithm to execute a predetermined PCR cycle. A 12 V DC fan aided in the active cooling of the PCR mixture. The fluorescence signal from the PCR mixture was excited using a white LED (MWWHL4, Thorlabs, Inc., Newton, New Jersey) with a bandpass filter in the range of 460–487 nm (FF01-474/27-25, Semrock, Rochester, New York, USA) and was captured by a CMOS camera (puA1600-60um, Basler AG, Ahrensburg, Germany), preceded by a 512–557 nm bandpass filter (FF01-525/45-25, Semrock, Rochester, New York, USA). The temperature profile of the PCR mixture, along with the timing of the control signals for the operation of the photothermal light source, cooling fan, and fluorescent excitation LED in the photothermal PCR setup, is depicted in Fig. 1c. The PCR amplification plot was derived from the average pixel intensity of the fluorescence signal recorded during the annealing and extension step in each cycle. The entire photothermal PCR process was controlled and monitored using the LabVIEW program.

### PCR mixtures and electrophoretic imaging

We prepared two different PCR mixtures for photothermal PCR and photothermal qPCR. PCR mixtures for photothermal PCR and photothermal q-PCR were prepared as indicated in Tables 1 and 2, respectively. Au nanoshells (GSGN800, nanoComposix, San Diego, California, USA), possessing a total diameter of 155 ± 7 nm and a core diameter of 121 ± 5 nm, were utilized (Supplementary Fig. S3). The surface of the Au nanoshell was functionalized with mPEG 5 kDa. A strong absorption peak was observed around the 800 nm wavelength, as shown in Fig. 1b and Supplementary Fig. S3. Both PCRs were targeted on the lambda DNA (Merck KGaA, Darmstadt, Germany). For photothermal PCR, Z-Taq polymerase, 10X Z-Taq Buffer, and dNTP Mixture were used (Takara Korea Biomedical Inc., Seoul, Korea). The sequences shown in the table were synthesized by Bioneer (Daejeon, South Korea). Bovine serum albumin (New England Biolabs, Ipswich, MA, USA) was used for relieving interference in PCR ^27^. For photothermal q-PCR, 20X TaqMan gene assay and 2X TaqMan™ Fast Advanced Master Mix from Applied Biosystems (Waltham, MA, USA) were used. The 10 μL of each PCR mixture was placed into 0.2 μL PCR tube for photothermal PCR and then covered with 30 μL of mineral oil to prevent evaporation during thermal cycling. After amplification, a mixture of 10 μL of PCR product and 2 μL of DNA Gel Loading Dye (6X) (Thermo Fisher Scientific Inc., Waltham, MA, USA) was loaded onto 1.5% agarose gels with SYBR Safe DNA gel stain (Invitrogen, Waltham, MA, USA) and run in a gel electrophoresis (Mupid-exU; Takara Korea Biomedical, Seoul, South Korea) at 50 V for 40 min. A 100 base pair DNA ladder (Bioneer, Daejeon, South Korea) was used to confirm the size of product. The quantification of electrophoretic results, aimed at determining the optimal PCR cycle conditions, was based on the total intensity of bands at 98 base pairs for each lane in the electrophoretic images by using ImageJ software.

**Table 1:** Composition of PCR mixture for photothermal PCR.

Components	Volume (μL)
10X Z-taq Buffer (Mg^2+^ plus) (30 mM)	1
Takara Z-Taq (2.5 U/μL)	0.1
dNTP mixture (2.5 mM each)	0.8
Lambda forward primer (10 μM)	0.9
Lambda reverse primer (10 μM)	0.9
Bovine serum albumin (20 μg/μL)	0.5
Lambda DNA (0.5 ng/μL)	2
Gold nanoshells (1.53 μg/μL)	1
Ultra pure water	2.8

**Table 2:** Composition of PCR mixture for photothermal qPCR.

Components	Volume (μL)
20X TaqMan™ gene expression assay, FAM^a^	0.5
2X TaqMan™ fast advanced master Mix	5
Bovine serum albumin	0.25
Lambda DNA (0.5 ng/μl)	1
Gold nanoshells (1.53 μg/μl)	1
Ultra pure water	2.25

^a^Pre-formulated assay consists of two primers and one Taqman probe as shown below.

Forward and reverse primers (900 nM each final 1X concentration).

Taqman probe: FAM dye-labeled TaqMan minor groove binder probe (250 nM final 1X concentration).

### Electromagnetic simulation

For the electromagnetic simulation, finite element method (FEM) simulation was utilized using commercially-available software (COMSOL Multiphysics 6.0, Wave Optics Module). Studies were conducted in 3D within the wavelength domain. The simulation domain consisted of a gold nanoshell and a water domain, truncated by a perfectly matched layer (PML). Additionally, a scattering boundary condition was implemented to eliminate any undesirable scattering effects at the domain boundaries. The core diameter and the shell thickness of the nanoparticle were assumed to be 120 nm and 17 nm, respectively. The complex refractive index of gold was obtained from the data of Johnson and Christy^28^, while the refractive index of silicon dioxide (SiO_2_) was acquired from the data of Malitson^29^. The refractive index of water was assumed to be 1.33. In order to calculate the extinction spectra of the gold nanoshell, absorption ${\sigma }_{abs}$ and scattering ${\sigma }_{sca}$ cross-sections were first determined. ${\sigma }_{abs}$ and ${\sigma }_{sca}$ are defined as

$${\sigma }_{abs}=\frac{{W}_{abs}}{{P}_{inc}}, {\sigma }_{sca}=\frac{{W}_{sca}}{{P}_{inc}}$$

where ${P}_{inc}, {W}_{abs},{W}_{sca}$ are incident irradiance, energy rate absorbed by the gold nanoshell, and scattered energy rate, respectively. The extinction cross-section ${\sigma }_{ext}$ can be ascertained as the summation of the absorption and scattering cross-section, i.e. ${\sigma }_{ext}={\sigma }_{abs}+{\sigma }_{sca}$.

## Results and discussion

**Figure 2:**
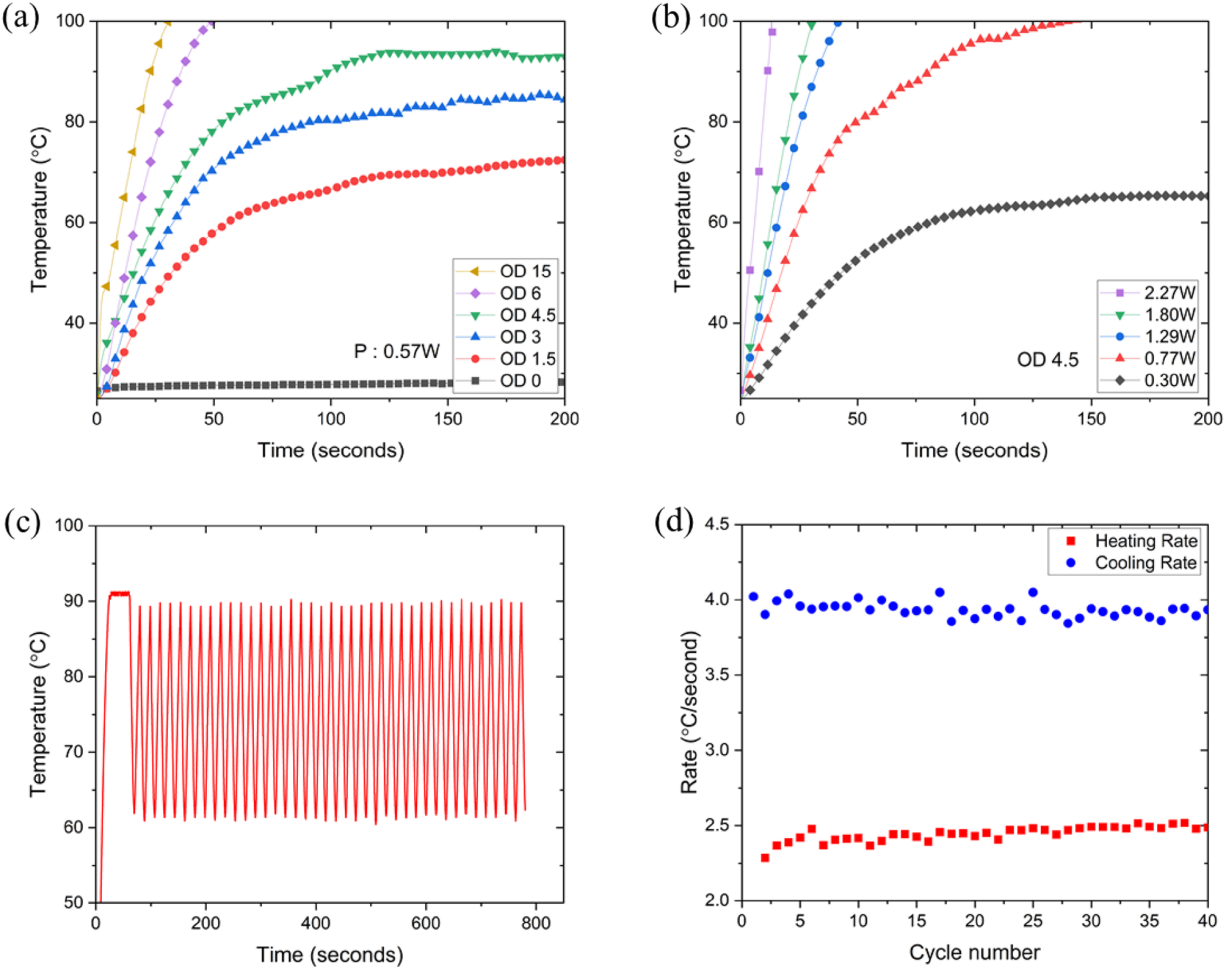
Chracterization of photothermal properties of Au nanoshell solution and dynamics of photothermal PCR using Au nanoshell solutions. (**a**) Photothermal heat generation in Au nanoshell solutions with varying the nanoshell concentrations occurs under the irradiation of a 0.57 W, 808 nm laser. (**b**) Photothermal heat generation in Au nanoshell solutions occurs with varying irradiated optical power from an 808 nm laser at a nanoshell concentration of OD 4.5. (**c**) A representative temperature profile for photothermal PCR includes a denaturation step at 91 °C and an annealing/extension step at 60 °C, spanning a total of 40 cycles. The entire process takes less than 800 s, which includes 60 s for pre-denaturation. (**d**) The ramping rate for photothermal heating is ~ 2.4 °C/s (red square) and the cooling rate is ~ 3.9 °C/s (blue circle), which remains almost identical over whole photothermal PCR cycle.

Figure 2a,b display the photothermal heating effect on solutions with varying concentrations of Au nanoshells and different optical outputs from the photothermal light source, respectively. Upon continuous laser irradiation at 0.57 W, the temperature of the Au nanoshell solutions with optical densities (OD) of 1.5, 3, and 4.5 stabilized at equilibrium temperatures of 72, 85, and 93 °C, respectively. Meanwhile, Solutions with OD 6 and OD 15 reached temperatures exceeding 100 °C within 49 and 30 s, respectively, under the same laser power (Fig. 2a). A higher concentration of nanoshells results in a larger temperature increase from the photothermal effect. Notably, solutions without nanoshells show negligible temperature change upon light irradiation (Fig. 2a, black line). Upon laser irradiation at a power of 0.3 W, the Au nanoshell solution with an optical density (OD) of 4.5 stabilized at an equilibrium temperature of 65 °C. When the laser power was increased to 0.77 W, 1.29 W, 1.80 W, and 2.27 W, the temperatures of the solution surpassed 100 °C in 142, 42, 30, and 14 s, respectively (Fig. 2b). As the optical output from the photothermal source increases, the temperature rise becomes both larger and more rapid. Based on these findings, it is evident that for the PCR mixture temperature to surpass the denaturation temperature, the nanoshell concentration must exceed a certain threshold of OD 4.5. For rapid thermal cycling, a photothermal light source requires a laser with an output power of over 1.5 watts (Fig. 2b, green line). Thus, both the nanoshell concentration and the optical output must surpass specific values to establish optimal conditions for photothermal PCR.

We established the conditions for photothermal PCR with an OD of 4.5 and an optical output of 1.5 W, corresponding to the Au nanoshell concentration and optical output, respectively. Under these conditions, a 2-step photothermal PCR cycle was conducted, alternating between 91 °C for denaturation and 60 °C for annealing and extension. Figure 2c shows the temperature profile of the PCR mixture during 40 photothermal PCR cycles. The heating rate and cooling rate of the photothermal PCR cycle were calculated as the temperature difference divided by the time taken for the heating and cooling processes, as depicted in Fig. 2d. The total time for 40 photothermal PCR cycles was 800 s, with the heating and cooling rates of 2.4 ± 0.049 °C/second and 3.9 ± 0.052 °C/s, respectively.

**Figure 3:**
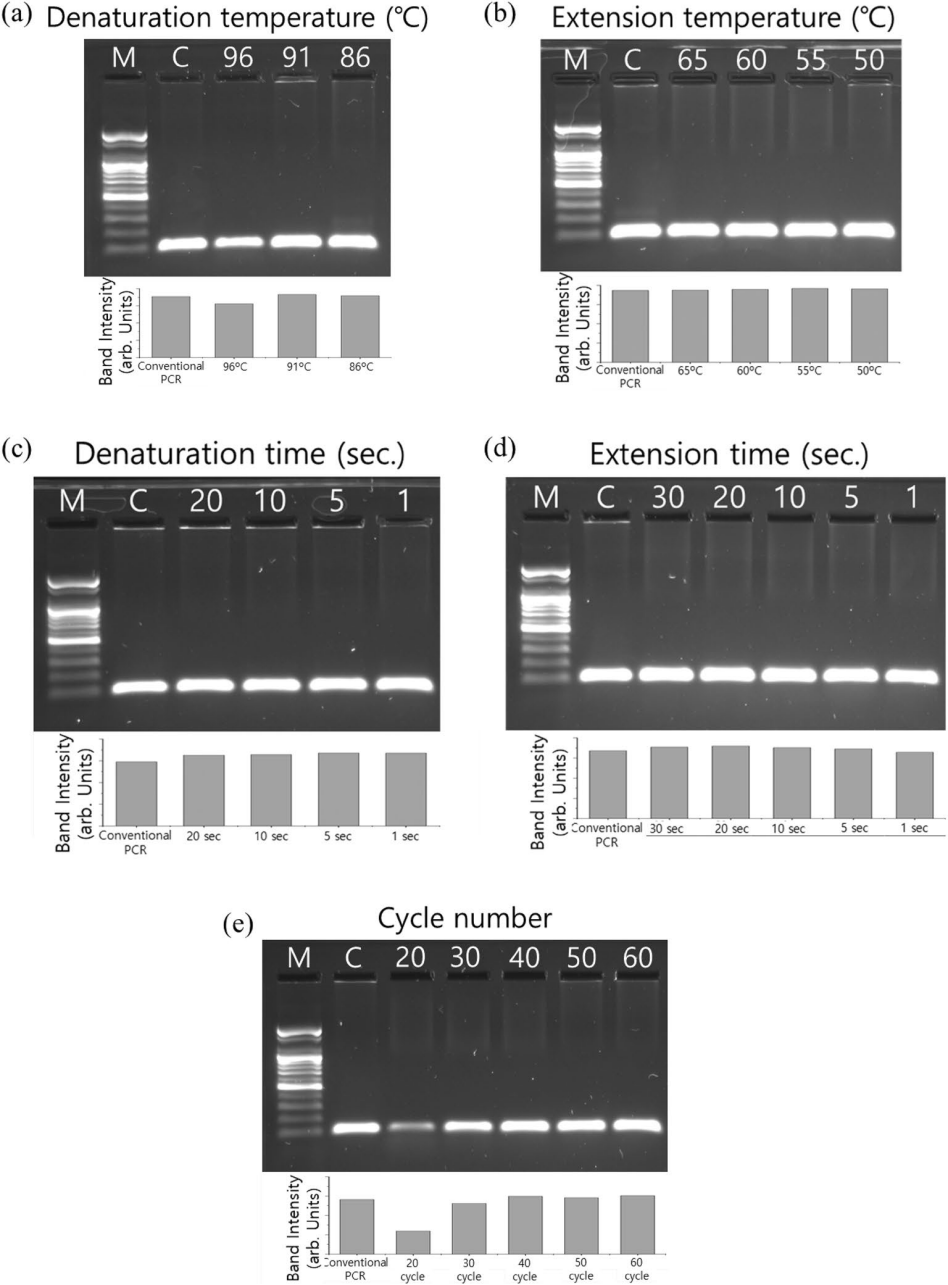
Optimization of photothermal PCR conditions. 1.5% agarose gel electrophoresis of amplicons from photothermal PCR performed with different denaturation and annealing/extension temperatures, times, and cycle numbers. Amplicons from photothermal PCR with (**a**) different denaturation temperatures (**b**) different annealing/extension temperatures (**c**) different denaturation times (**d**) different annealing/extension times (**e**) different cycle number. The gel images in (**a**)–(**e**) are cropped. The original gel images are presented in Supplementary Fig. S4–S8. Lane M: 100 bp DNA Ladder, Lane C: conventional PCR.

We employed the PCR mixture outlined in Table 1 to evaluate the efficacy of photothermal PCR for DNA amplification. Initially, DNA amplification results from photothermal PCR were confirmed through electrophoretic imaging. Alongside these images, graphs illustrating the variations in band intensities observed in the electrophoretic images were presented. Our photothermal PCR approach produced a distinct 98-bp band, as seen in the electrophoretic images (Fig. 3a–e), confirming the successful amplification of λ-DNA. We adjusted various cycle condition parameters to ascertain the optimal photothermal PCR cycle conditions. Figure 3a,b show the PCR amplification outcomes when modulating the denaturation temperature, as well as the annealing/extension temperatures over a two-step, 60-cycle PCR process. The denaturation time was set at 20 s, while the annealing and extension time lasted for 30 s. The electrophoretic imaging revealed consistent band intensities at denaturation temperatures of 91 °C and 86 °C; however, a decrease in band intensity was observed at a denaturation temperature of 96 °C (Fig. 3a). Conversely, changes in the extension temperature, ranging from 65 to 50 °C, did not impact the band intensity (Fig. 3b). However, reduced band intensity was observed in the electrophoretic imaging under certain conditions, specifically when the denaturation temperature was set to 86 °C and the extension temperature to 65 °C (data not shown). Thus, we established that the optimal temperature range for a rapid two-step photothermal PCR cycle fluctuates between 91 and 60 °C. Subsequently, with the goal of reducing the photothermal PCR cycle duration, we explored the PCR amplification results concerning changes in the length of each stage. The denaturation time varied between 20 and 1 s, while the annealing and extension times ranged from 30 to 1 s. The findings, illustrated in Fig. 3c,d, suggest that reducing the duration of each phase to just 1 s does not alter the PCR amplification results. Additionally, we examined the PCR amplification outcomes when adjusting the number of PCR cycles, another pivotal variable influencing the total time. Our results indicate that at least 40 cycles are crucial for significant PCR amplification, as evidenced in Fig. 3e.

**Figure 4:**
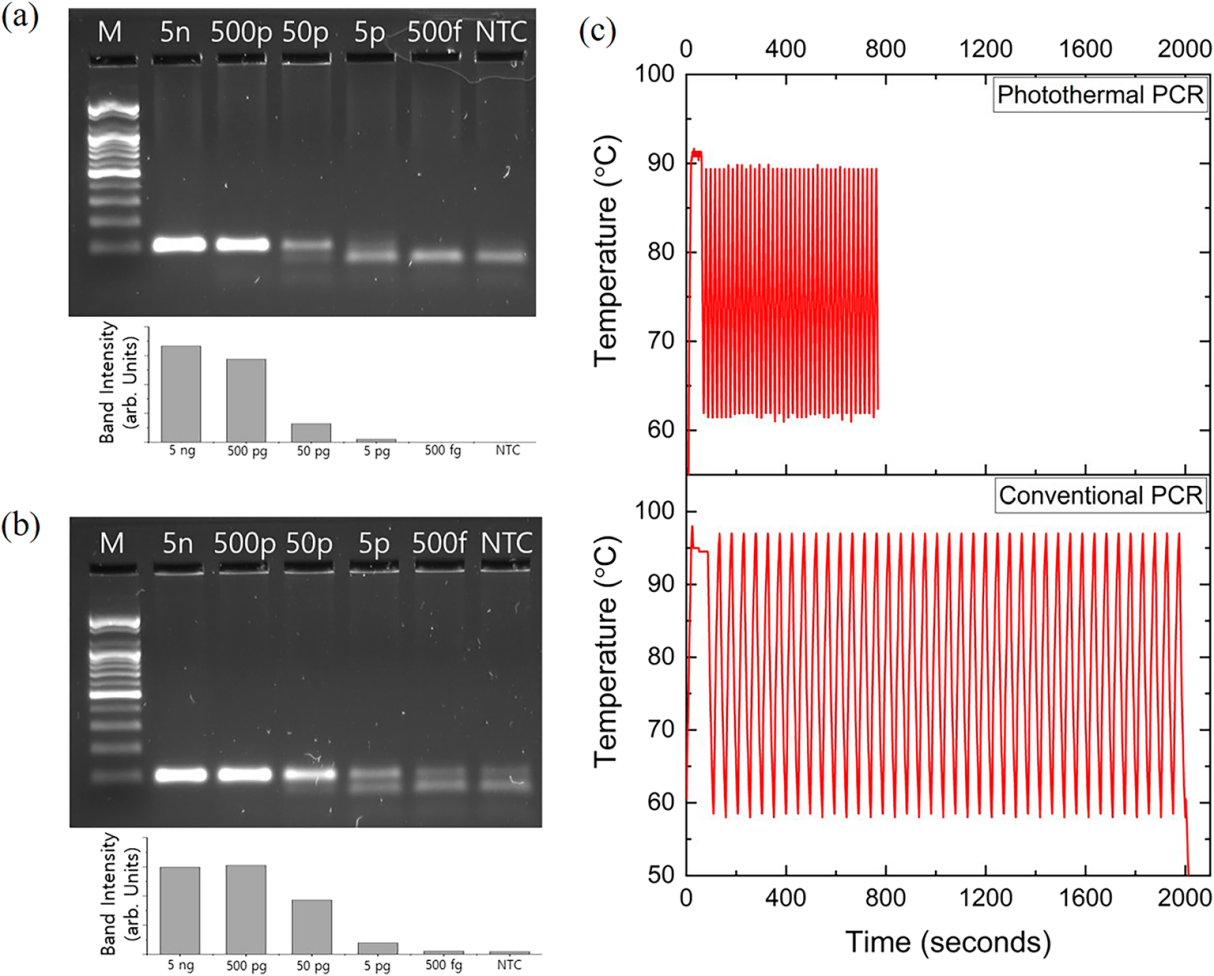
Electrophoretic image and band intensity graph showing DNA amplification results of PCR mixture containing 5 ng, 500 pg, 50 pg, 5 pg, and 500 fg of lambda DNA and NTC by (**a**) photothermal PCR and (**b**) conventional PCR. (**c**) Temperature profiles of the PCR mixture during the progression of the 40-cycle, two-step photothermal PCR (top) and the conventional PCR (bottom) process. Notably, the photothermal PCR completes its analysis in just 800 s, in contrast to the conventional PCR, which requires 2000 s. This represents a significant reduction in analysis time by 60%, demonstrating the efficiency of the photothermal PCR method. The gel images in (**a**) and (**b**) are cropped. The original gel images are presented in Supplementary Figs. S9 and S10. Lane M: 100 bp DNA Ladder, Lane NTC : negative template control.

To evaluate the detection sensitivity of photothermal PCR, PCR mixtures with varying amounts of template DNA were prepared to determine the limit of detection (LoD) for DNA amplification. PCR mixtures containing lambda DNA in quantities of 5 ng, 500 pg, 50 pg, 5 pg, and 500 fg, along with negative control samples, were subjected to photothermal PCR. For LoD comparison, identical PCR mixtures were amplified using both photothermal PCR and conventional PCR equipment (SimpliAmp, Thermo Fisher Scientific Inc., Waltham, MA, USA). The amplification involved a 40-cycle process: photothermal PCR at 91 °C for 1 s and 60 °C for 1 s, and conventional PCR at 94 °C for 1 s and 62 °C for 1 s, with DNA amplification confirmed via electrophoretic imaging. Figure 4a,b display the electrophoretic images and intensity graphs for DNA amplification from photothermal PCR and conventional PCR, respectively. Mixtures with 5 ng and 500 pg of lambda DNA showed similar DNA amplification results in both methods. However, PCR mixtures with 50 pg of lambda DNA exhibited different amplification levels in photothermal and conventional PCR. Although photothermal PCR amplified a mixture with 50 pg of lambda DNA, the band intensity in electrophoresis was weaker compared to conventional PCR. Figure 4c compares the 40-cycle temperature profiles of both methods, highlighting that photothermal PCR reduces the total analysis time by 60% compared to conventional PCR. This suggests that while photothermal PCR’s LoD is slightly higher than conventional PCR, its significant advantage lies in markedly reducing analysis time.

**Figure 5:**
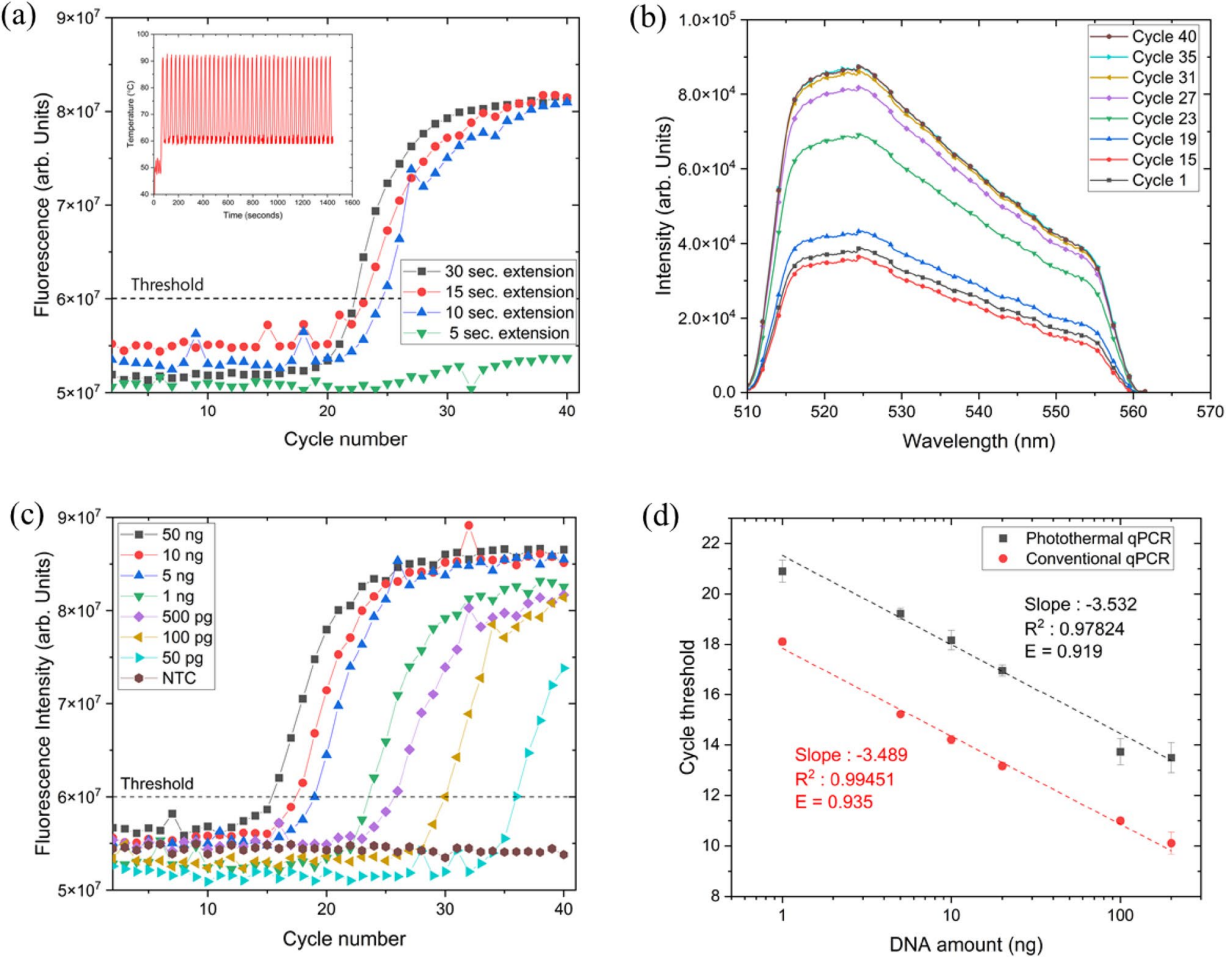
(**a**) Amplification curves for PCR mixtures containing 1 ng of lambda DNA using photothermal qPCR. The extension time within the photothermal qPCR was varied, ranging from 30 s down to 1 s. Inset : Temperature profile of a 40-cycle photothermal qPCR process performed with a denaturation time of 5 s and annealing/extension time of 15 s. (**b**) Fluorescence emission spectrum of the PCR mixture at each cycle of the phothermal qPCR. The emission spectrum falls within the spectrum range of the FAM-labeled Taqman probe. (**c**) Amplification curves of a 40-cycle photothermal qPCR amplification using PCR mixtures, where the amounts of lambda DNA were varied from 50 nanograms down to 50 picograms. (**d**) Standard curve for photothermal qPCR, which illustrates the relationship between the threshold cycle value and the initial template DNA amount, in comparison to the standard curve for conventional qPCR.

Next, we enhanced the photothermal PCR equipment to facilitate immediate verification of DNA amplification in real time. We carried out photothermal quantitative PCR by measuring the fluorescence from the PCR mixture that incorporated a FAM-labeled Taqman probe (refer to Table 2) at each PCR cycles. The Au nanoshell concentration in the PCR mixture was set at OD 4.5. This specific concentration is based on the findings that a higher Au nanoshell concentration inversely affects the fluorescence intensity due to the quenching effect of the Au nanoshell^6^ (Supplementary Fig. S11). The PCR mixture for photothermal qPCR (refer to Table 2) includes Taq polymerase, which generally requires approximately one minute per kilobase (kb) of amplicon for full extension^30,31^. Consequently, the extension duration for photothermal qPCR should be prolonged relative to that of photothermal PCR, which employs Takara Z-Taq. The latter offers a significantly faster throughput, with an annealing/extension time of just 10–20 s per kb, over five times quicker than the rate achieved with Taq polymerase. Amplification curves for PCR mixtures with 1 ng of lambda DNA were examined by modulating the extension time within the PCR cycle, ranging from 30 to 1 s. As depicted in Fig. 5a, the threshold cycle (C_t_) values of the PCR mixtures for extension times of 30 (C_t_ = 22.26) and 15 s (C_t_ = 22.85) were nearly equivalent. Conversely, extension time of 10 (C_t_ = 24.48) seconds experienced a decline in PCR amplification efficiency, as evidenced by an increased C_t_ value. The C_t_ value was determined as the cycle number at which the PCR amplification curve intersects the threshold of fluorescence intensity. We established the optimal PCR cycle conditions for the photothermal qPCR, setting a denaturation temperature of 91 °C for 5 s, and an annealing/extension temperature of 60 °C for 15 s (Supplementary Fig. S12). The cycle conditions for conventional qPCR (QuantStudio 3, Thermo Fisher Scientific Inc., Waltham, MA, USA) were established with a denaturation temperature of 91 °C for 15 s, followed by an annealing/extension phase at 60 °C for 60 s. We measured the fluorescence emission spectrum of the PCR mixture at each cycle (Fig. 5b). We confirmed that the fluorescence emission spectrum we measured falls within the spectrum range of the FAM-labeled Taqman probe. The fluorescence spectral intensity increased in accordance with the trend of the PCR amplification curve in Fig. 5a. To evaluate the sensitivity and linearity of photothermal qPCR, we monitored the variation in C_t_ values in photothermal qPCR amplification outcomes in relation to the quantity of template DNA in the PCR mixture. Figure 5c shows the results of a 40-cycle photothermal qPCR amplification using PCR mixtures, where the amounts of lambda DNA were varied from 50 nanograms down to 50 picograms. Significant amplification was observed, with the threshold cycle occurring within 30 cycles in PCR mixtures containing as little as 100 picogram of lambda DNA. Figure 5d presents a standard curve depicting the relationship between the C_t_ value and the initial quantity of template DNA, plotted on a logarithmic scale. Figure 5d presents the standard curve for photothermal qPCR, which illustrates the relationship between the C_t_ value and the initial template DNA amount, in comparison to the standard curve for conventional qPCR. The photothermal qPCR results indicate a highly linear detection sensitivity, ranging from 200 nanograms to 1 nanogram of lambda DNA. When compared with the conventional qPCR, the C_t_ value in the photothermal qPCR is higher, exhibiting an increase of 3–5 units. Additionally, the slope of the standard curve in the photothermal qPCR, at − 3.532, is less steep compared to that of the conventional qPCR, which is − 3.489. This suggests a lower efficiency (E) in the PCR amplification process using the photothermal method. The reason behind the higher C_t_ values in the photothermal qPCR compared to the conventional qPCR lies in the methodology of determining C_t_ values. In the conventional qPCR, the C_t_ value is calculated by estimating changes in fluorescence signal intensity near the baseline through a fitting process. However, in the photothermal qPCR, the C_t_ value is determined at the point where the fluorescence signal intensity distinctly surpasses a predefined threshold, clearly separated from the baseline. This methodological difference accounts for the increased C_t_ values observed in the photothermal qPCR. The reduced PCR amplification efficiency observed in photothermal qPCR, in comparison to conventional qPCR, is likely due to the less uniform temperature distribution within the PCR mixture during the photothermal process. The design of current photothermal qPCR equipment involves the use of a laser light source for photothermal heating, which is directed from below the reaction mixture. This setup results in the Au nanoshells, which are integral to the photothermal process, predominantly absorbing light energy at the bottom of the PCR tube. Although convection aids in heat circulation, it doesn’t entirely compensate for this uneven distribution. As a result, the upper part of the PCR mixture tends to be cooler than the lower part, which is thought to contribute to the reduced amplification efficiency observed in photothermal qPCR. Based on the findings, it appears that the photothermal qPCR, in its current implementation, exhibits relatively lower amplification efficiency but delivers a faster analysis process when compared to the conventional qPCR equipment. It is noteworthy that in PCR mixtures with Au nanoshell concentrations exceeding OD 15, the conventional qPCR equipment was unable to detect amplification. In contrast, the photothermal qPCR successfully identified a PCR amplification curve (Supplementary Fig. S13). This implies that by optimizing the photothermal qPCR protocol, the analysis time could be considerably reduced compared to that of conventional qPCR.

## Conclusion

Our study presented a novel photothermal quantitative PCR (qPCR) approach utilizing Au nanoshells for rapid and sensitive DNA amplification. This innovative approach demonstrated the potential for rapid and efficient DNA amplification, particularly notable in the photothermal PCR process where significant time reduction was achieved compared to conventional methods. The study meticulously calibrated and optimized the PCR conditions, including temperature profiles, cycle durations, and Au nanoshell concentrations, to attain optimal performance. Specifically, the photothermal PCR process achieved a significant reduction in total PCR time, with the complete cycle being completed in just 800 s, compared to the 2000s typically required by conventional PCR methods. This reduction in time is attributed to the optimized heating and cooling rates of the PCR process, which were measured to be 2.4 °C/s for heating and 3.9 °C/s for cooling. Additionally, the use of non-contact temperature monitoring and precise control mechanisms ensured accurate thermal regulation without lowering the PCR efficiency. This study also marks a pivotal advancement in PCR technology through the integration of a fluorescence detection system into our photothermal PCR setup, effectively converting it into a qPCR system. A key achievement following this enhancement was our ability to rapidly confirm the amplification of 1 ng of lambda DNA in as little as 1400 s, showcasing the method's potential for swift and effective DNA analysis, which is especially valuable in time-sensitive testing scenarios. Nevertheless, when comparing our photothermal qPCR with conventional qPCR techniques, we noted a slight reduction in performance, specifically in the areas of C_t_ values and overall efficiency. We attribute these disparities mainly to the potential overestimation of C_t_ values in our fluorescence detection approach and the challenges associated with uneven temperature distribution in the PCR mixture. This issue, inherent in our current photothermal design, impacts the uniformity and consistency of PCR reactions, and is an area identified for future improvement and optimization. In conclusion, our photothermal quantitative PCR (qPCR) approach using Au nanoshells, by significantly reducing DNA amplification time and enhancing sensitivity, paves the way for future advancements in rapid molecular diagnostics and offers ample scope for further refinement in PCR technology.

### Supplementary Information

Supplementary Information.

## Data Availability

All experimental results have been included in the manuscript. The data can be made available upon reasonable request to the corresponding author.
