# Supplementary material for: Rapid quantitative PCR equipment using photothermal conversion of Au nanoshell
Source: Sci Rep. 2024 Feb 16;14:3895. doi: 10.1038/s41598-024-54406-0 (PMC10873297; doi:10.1038/s41598-024-54406-0)
Supplement: Supplementary file 1 — Supplementary Information. [file 41598_2024_54406_MOESM1_ESM.docx]

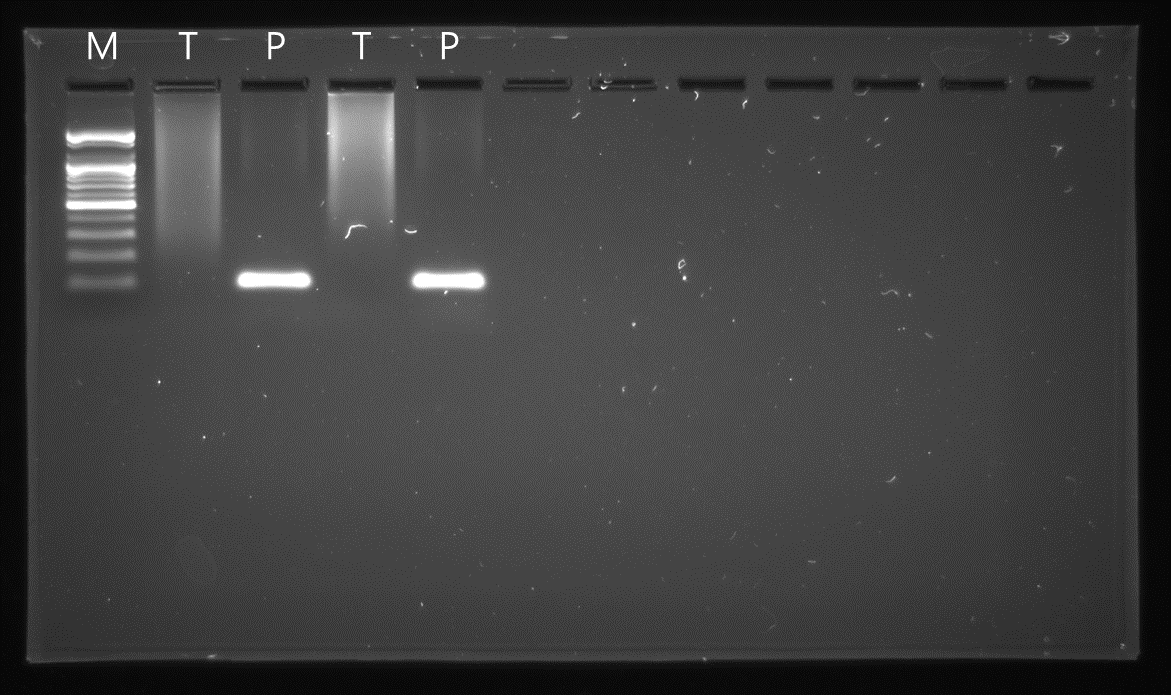


**Figure S1.** Inhibition of PCR by a contact K-type thermocouple. Photothermal PCR, where the temperature of the PCR mixture was measured using a non-contact pyrometer, resulted in a clear amplification band of lambda DNA. In contrast, using a contact K-type thermocouple for temperature measurement did not produce a distinct lambda DNA amplification band. Lane M : 100 bp DNA ladder, Lane T : thermocouple, Lane P : pyrometer


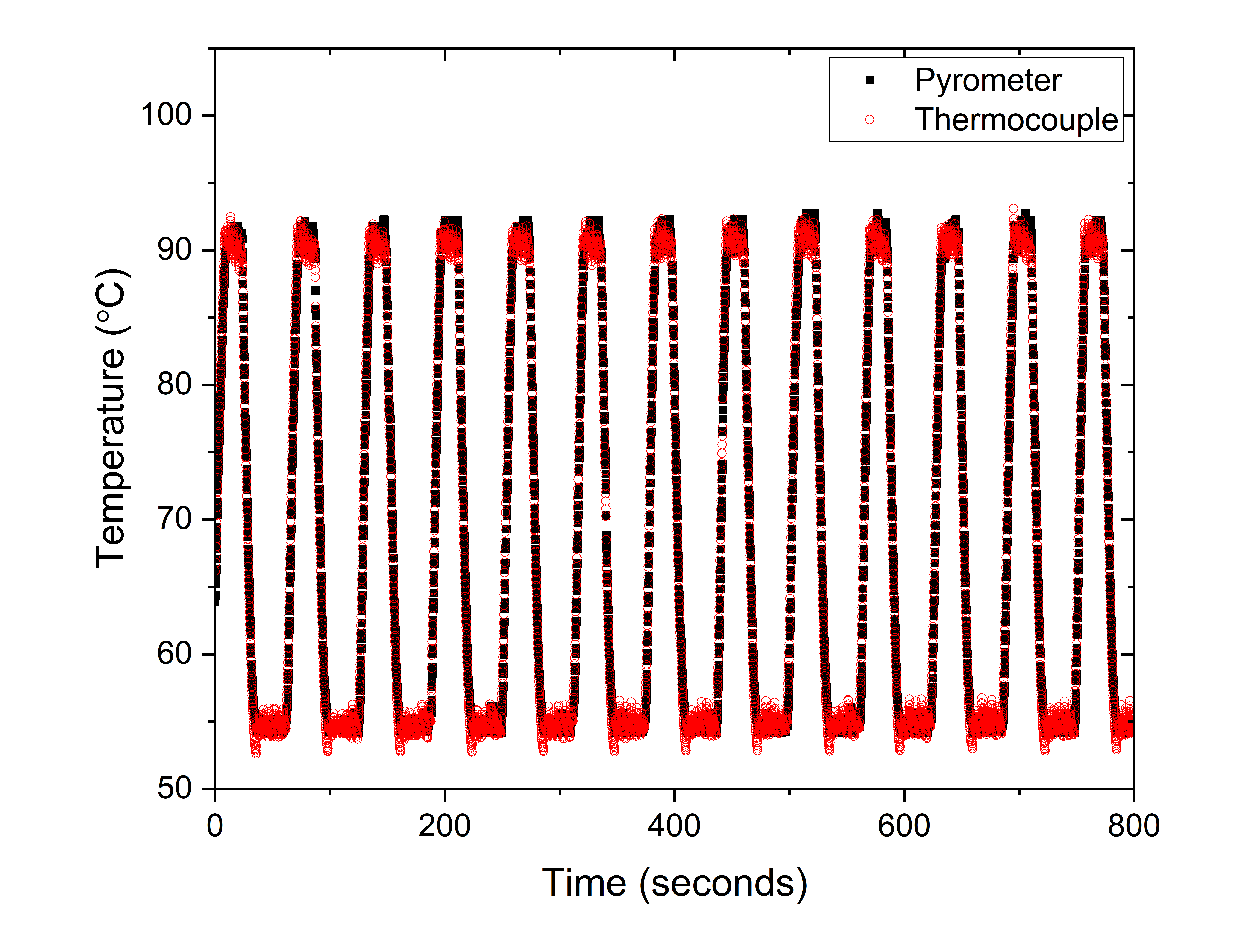


**Figure S2.** To calibrate the non-contact pyrometer's temperature, photothermal PCR was conducted with concurrent temperature measurements of the PCR mixture using both the pyrometer and a contact K-type thermocouple. The calibration process involved adjusting the pyrometer's emissivity settings until its temperature readings aligned with those from the thermocouple.


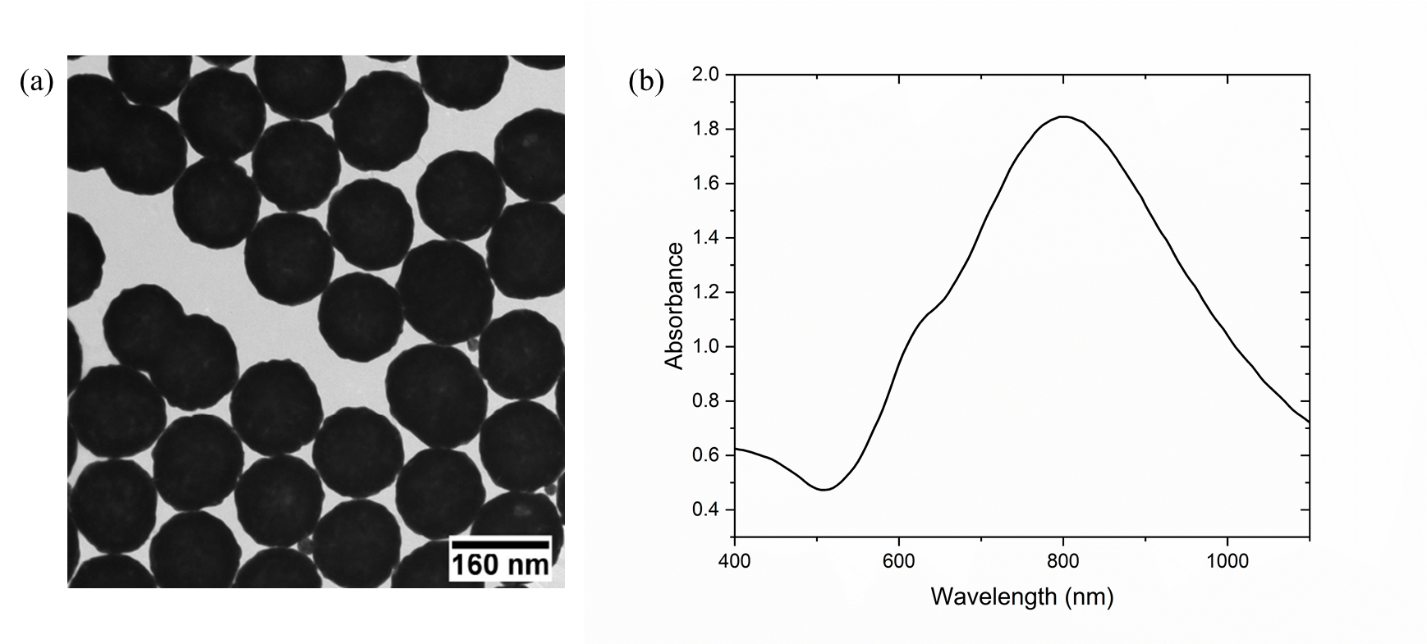


**Figure S3.** (a) TEM image of Au nanoshells (b) absorption spectrum of Au nanoshell solution.


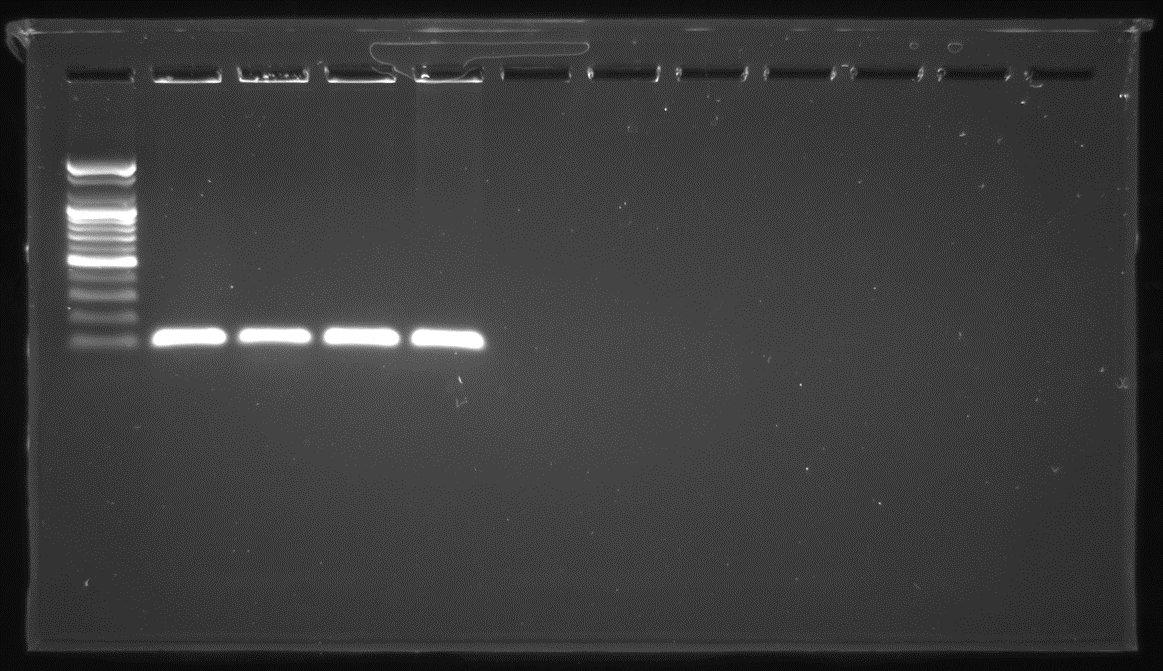


**Figure S4.** Original gel image of Figure 3(a).


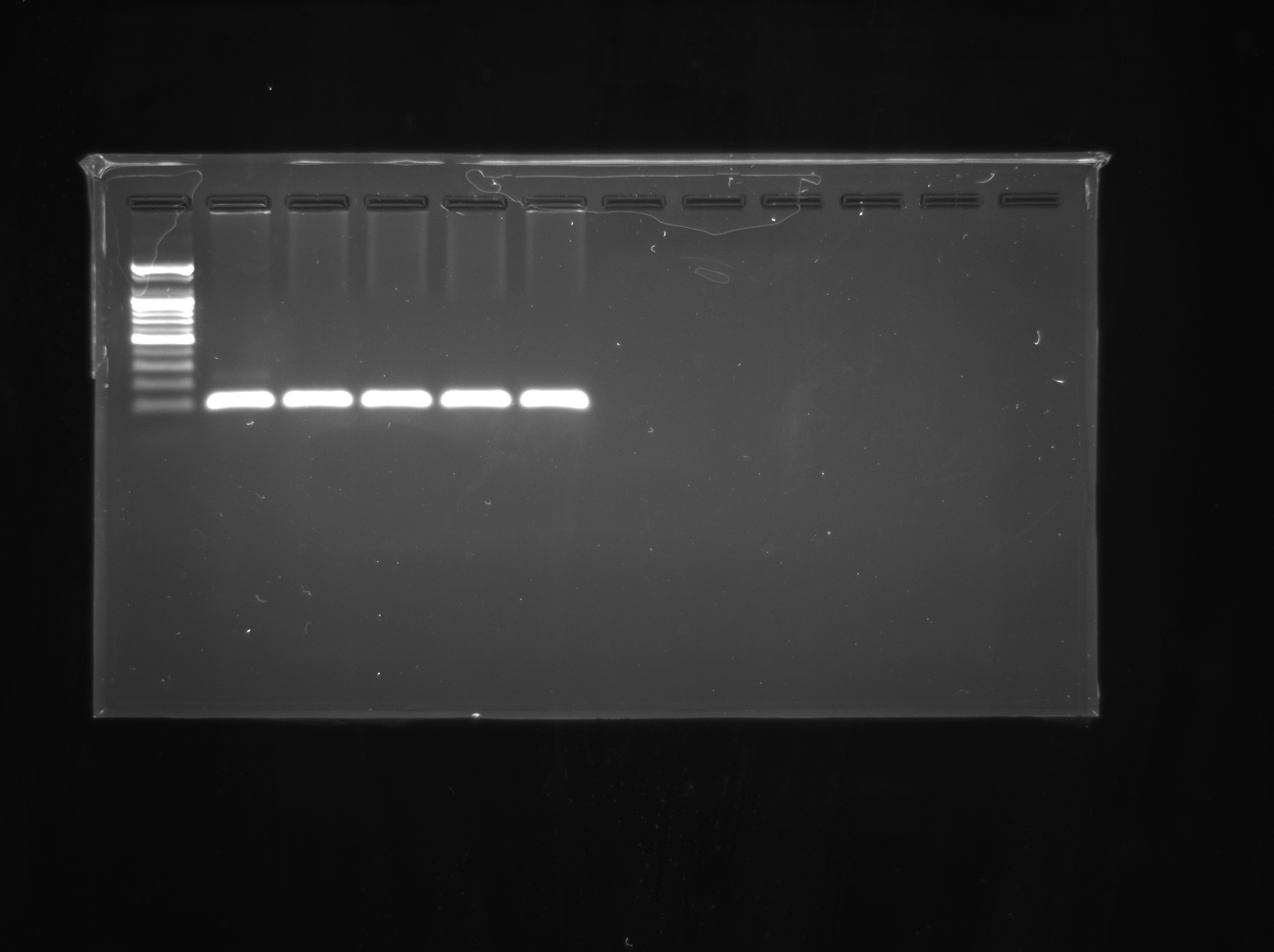


**Figure S5.** Original gel image of Figure 3(b).


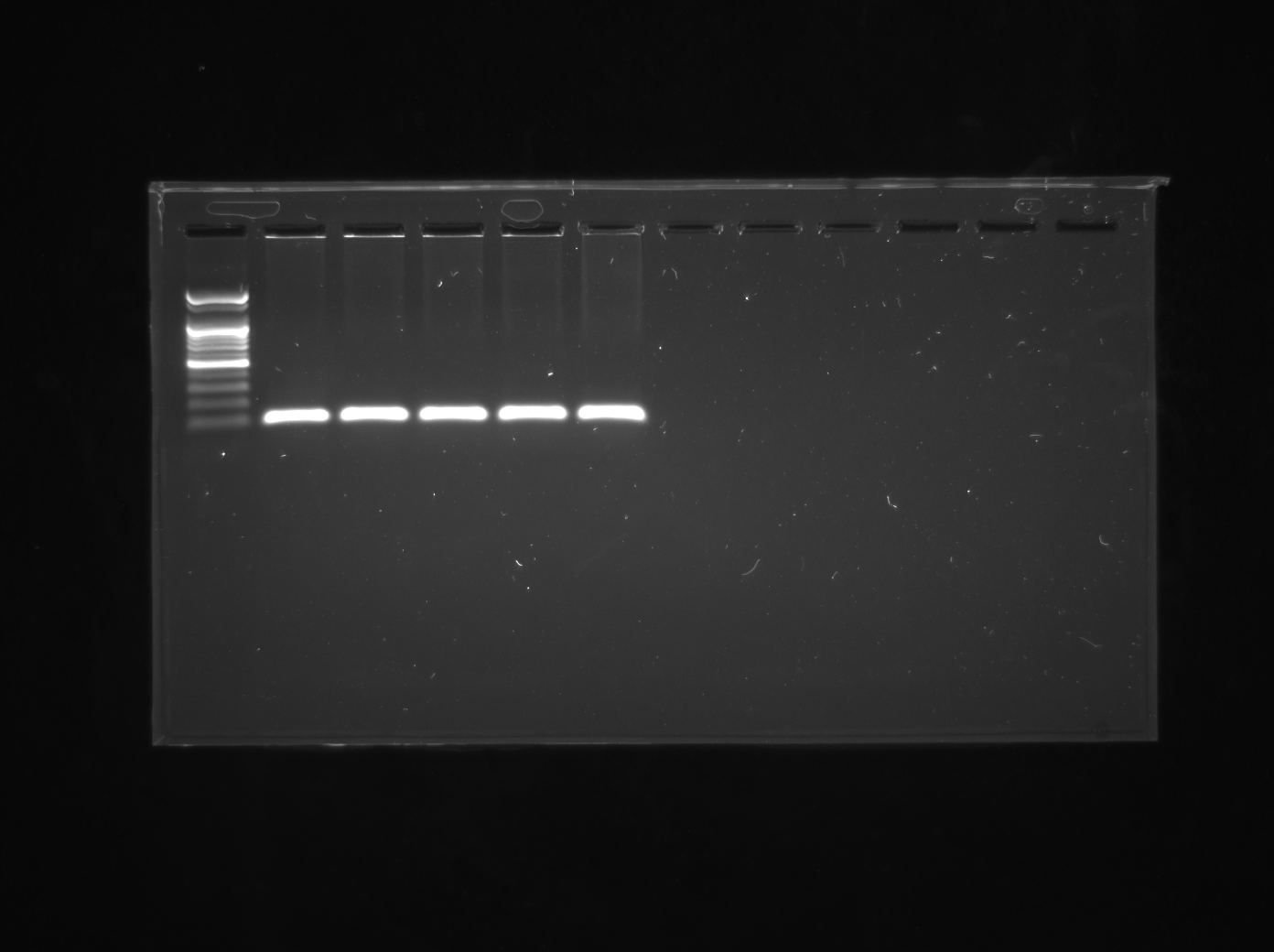


**Figure S6.** Original gel image of Figure 3(c).


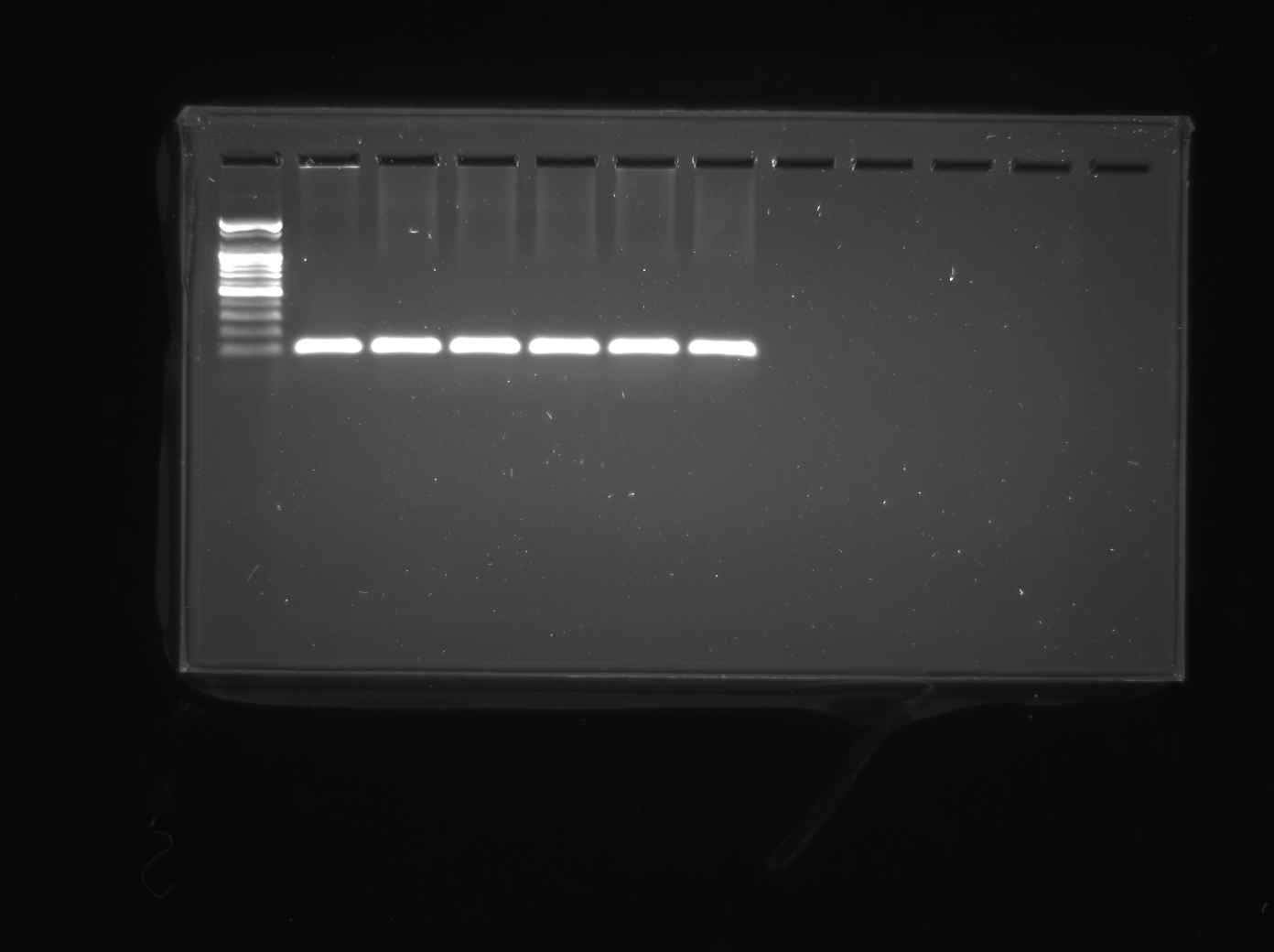


**Figure S7.** Original gel image of Figure 3(d).


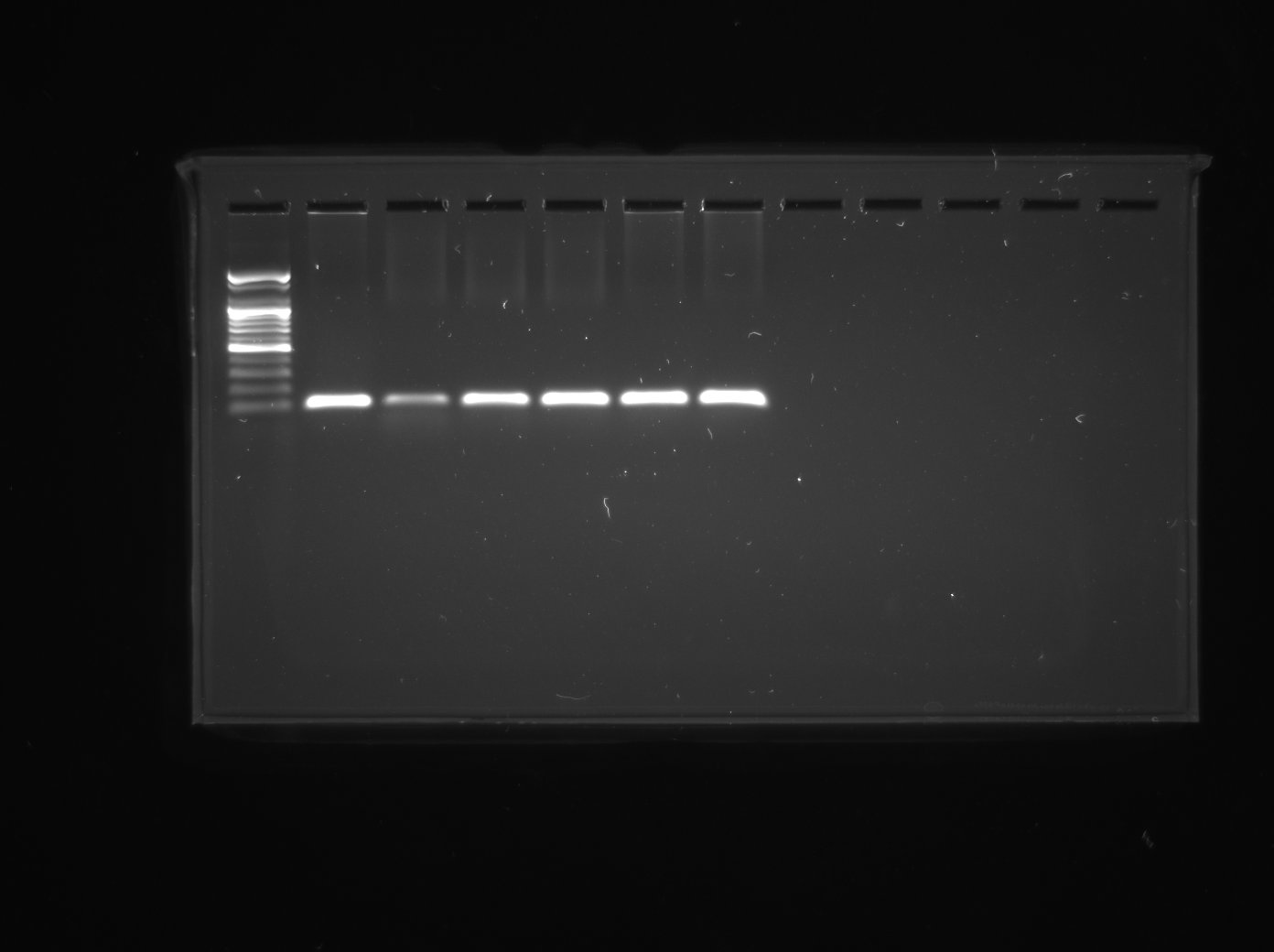


**Figure S8.** Original gel image of Figure 3(e).


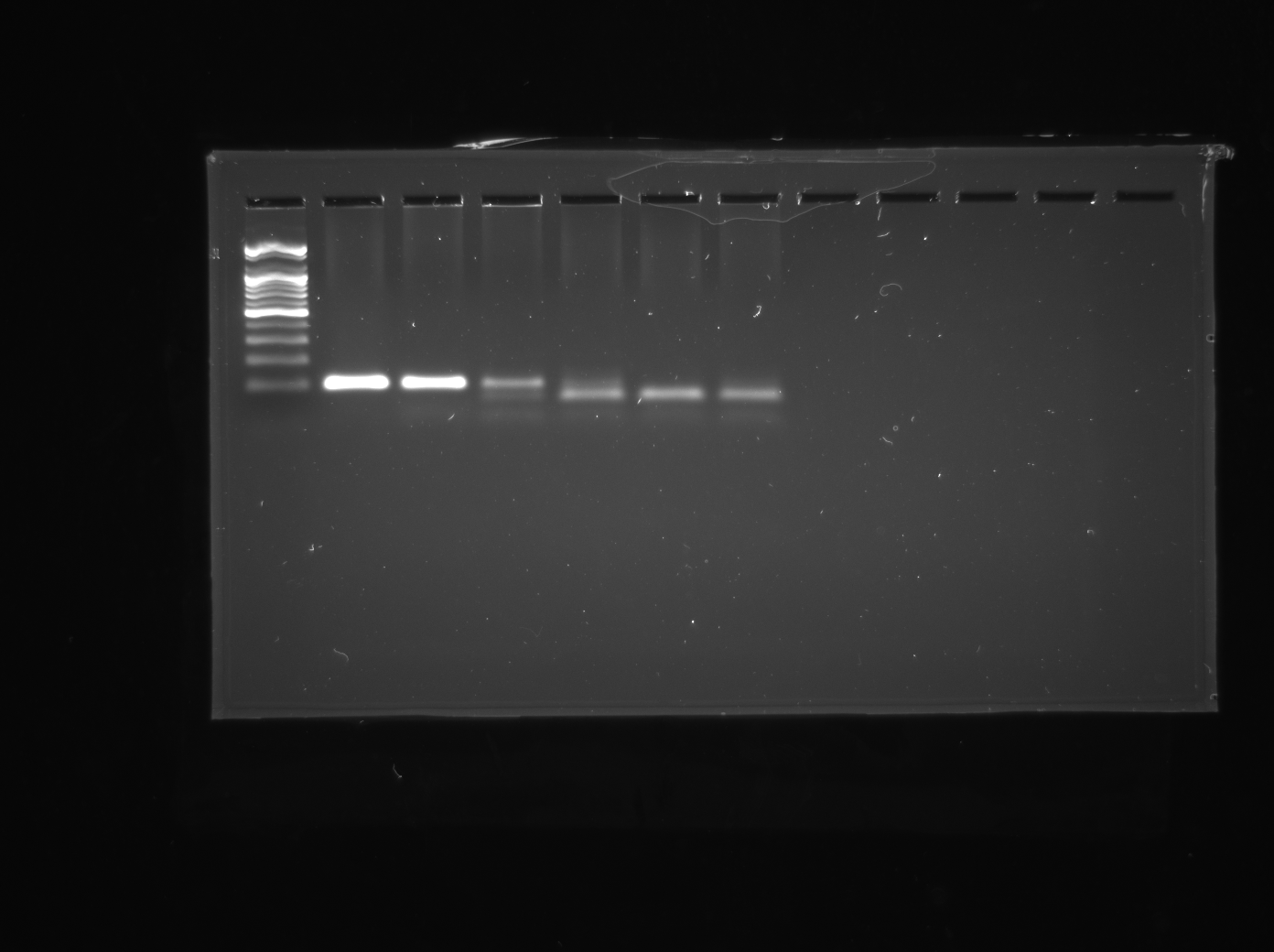


**Figure S9.** Original gel image of Figure 4(a).


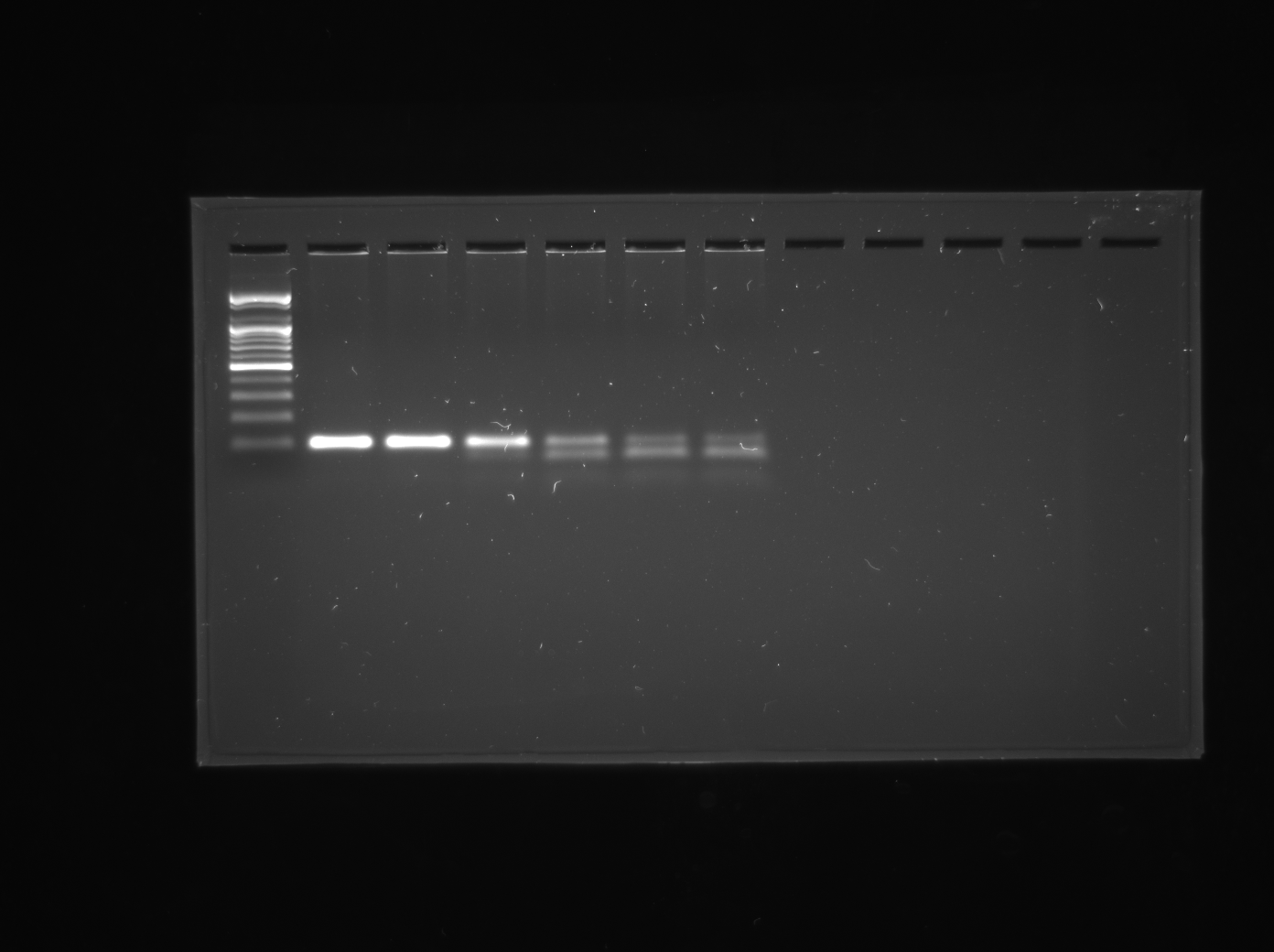


**Figure S10.** Original gel image of Figure 4(b).


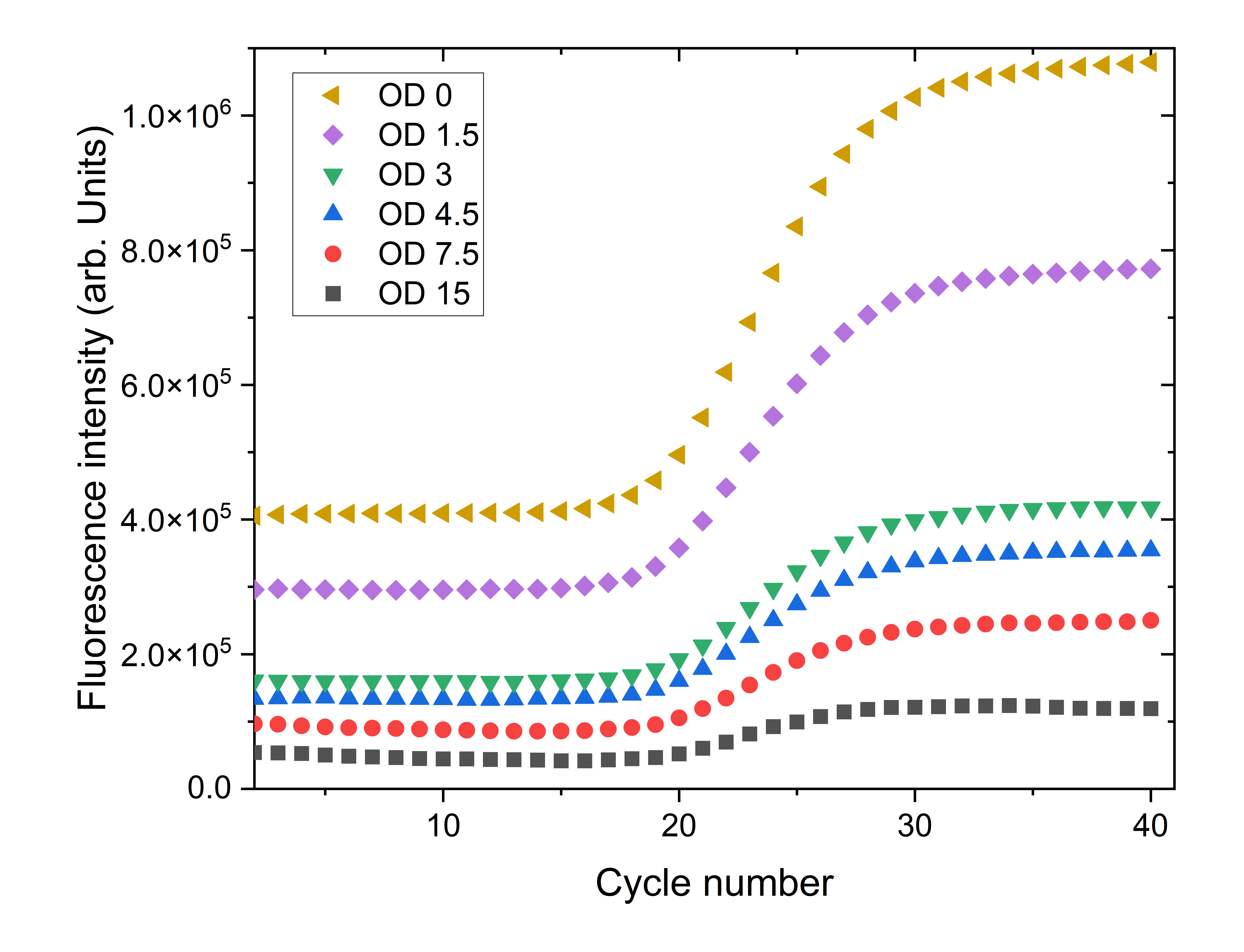


**Figure S11.** Increasing concentrations of gold nanoshells in the PCR mixture were found to correspond with a decrease in fluorescence signal intensity as measured by conventional qPCR equipment (Quantstudio 3, Thermo Fisher Scientific Inc., Waltham, MA, USA), indicative of the quenching effect of the nanoshells. This phenomenon was especially evident in samples with a gold nanoshell concentration at an optical density (OD) of 4.5, where the fluorescence signal intensity was approximately 40% compared to that in samples devoid of gold nanoshells.


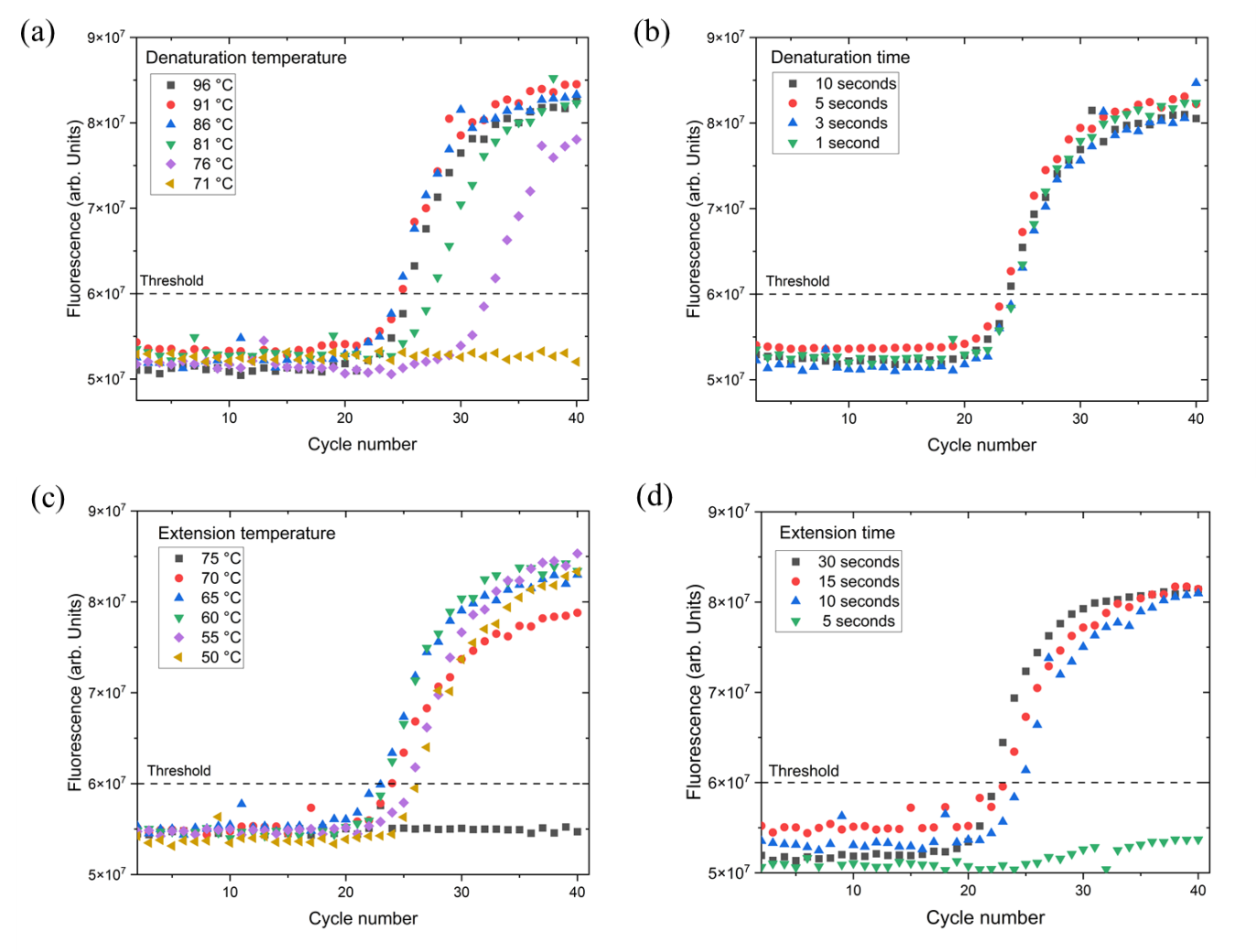


**Figure S12.** Variations in PCR amplification curves based on photothermal qPCR cycle conditions: (a) denaturation temperature, (b) denaturation time, (c) extension temperature, and (d) extension time.


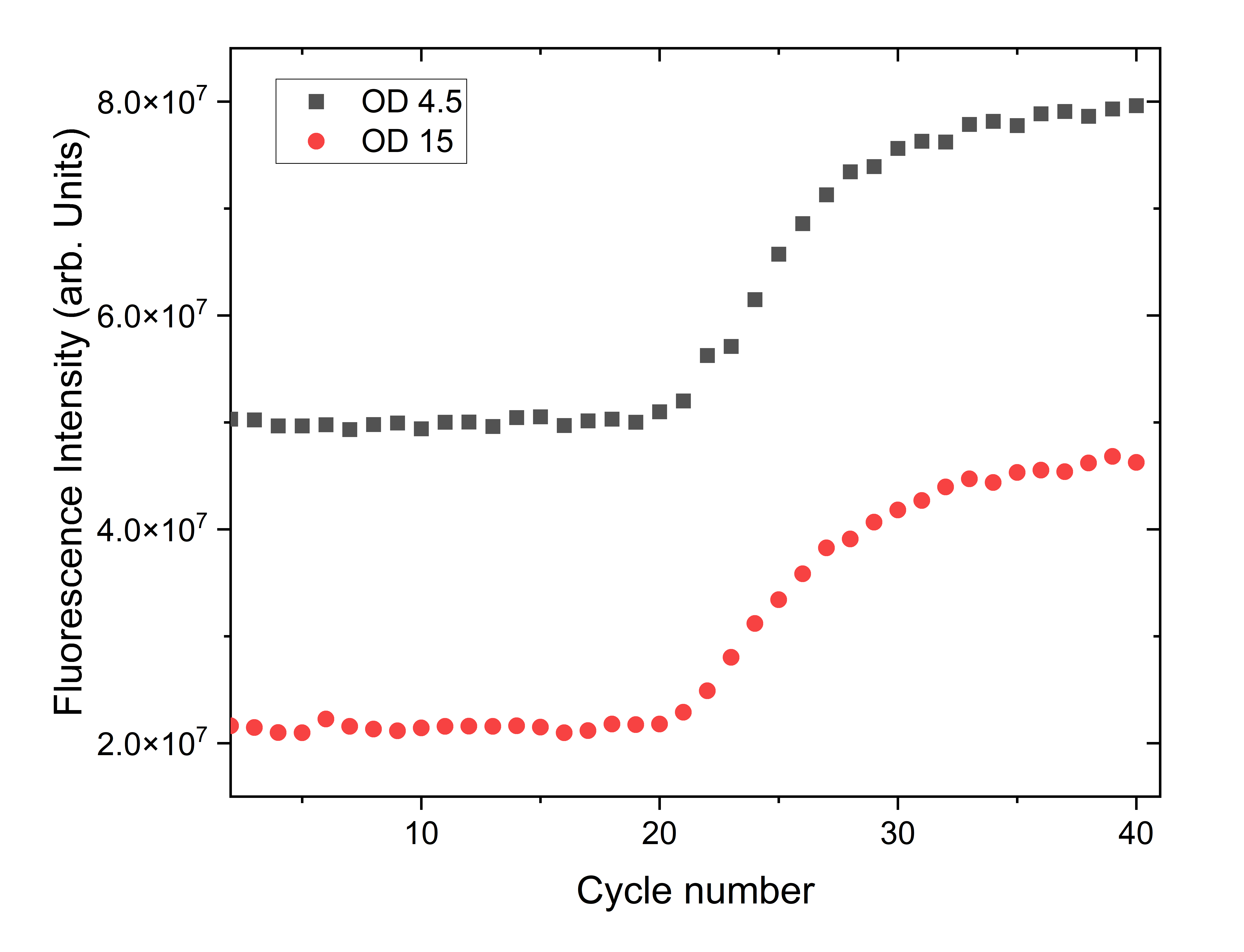


**Figure S13.** In photothermal PCR, samples containing a high concentration of gold nanoshells were effectively amplified, despite the lowered fluorescence signal intensity resulting from the nanoshells' quenching effect.

Table S1. Primers and probes used in this study

| Name | Sequence (5' to 3') |
| --- | --- |
| Lambda forward primer | CATCGTCTGCCTGTCATGGGCTGTTAAT |
| Lambda reverse primer | TCGCCAGCTTCAGTTCTCTGGCATTT |
| Taqman probe | TGATAACGCATTACCTACAAAGCCCAGCG |
